# Improved Metabolic Models for *E*. *coli* and *Mycoplasma genitalium* from GlobalFit, an Algorithm That Simultaneously Matches Growth and Non-Growth Data Sets

**DOI:** 10.1371/journal.pcbi.1005036

**Published:** 2016-08-02

**Authors:** Daniel Hartleb, Florian Jarre, Martin J. Lercher

**Affiliations:** 1 Institute for Computer Science and Cluster of Excellence on Plant Sciences, Heinrich Heine University, Düsseldorf, Germany; 2 Institute for Mathematics, Heinrich Heine University, Düsseldorf, Germany; EMBL-Heidelberg, GERMANY

## Abstract

Constraint-based metabolic modeling methods such as Flux Balance Analysis (FBA) are routinely used to predict the effects of genetic changes and to design strains with desired metabolic properties. The major bottleneck in modeling genome-scale metabolic systems is the establishment and manual curation of reliable stoichiometric models. Initial reconstructions are typically refined through comparisons to experimental growth data from gene knockouts or nutrient environments. Existing methods iteratively correct one erroneous model prediction at a time, resulting in accumulating network changes that are often not globally optimal. We present GlobalFit, a bi-level optimization method that finds a globally optimal network, by identifying the minimal set of network changes needed to correctly predict all experimentally observed growth and non-growth cases simultaneously. When applied to the genome-scale metabolic model of *Mycoplasma genitalium*, GlobalFit decreases unexplained gene knockout phenotypes by 79%, increasing accuracy from 87.3% (according to the current state-of-the-art) to 97.3%. While currently available computers do not allow a global optimization of the much larger metabolic network of *E*. coli, the main strengths of GlobalFit are already played out when considering only one growth and one non-growth case simultaneously. Application of a corresponding strategy halves the number of unexplained cases for the already highly curated *E*. *coli* model, increasing accuracy from 90.8% to 95.4%.

## Author Summary

Mathematical models that aim to describe the complete metabolism of a cell help us understand cellular metabolic capabilities and evolution, and aid the biotechnological design of microbial strains with desired properties. Draft models are frequently improved through adjustments that increase the agreement of growth/non-growth predictions with observations from gene knockout experiments. Automated methods for this task typically correct one erroneous prediction after the other. We present GlobalFit, a novel method that can consider all experiments and all possible changes simultaneously to identify model modifications that are globally optimal (i.e., that correct the largest possible number of wrong predictions while introducing sets of changes that are most compatible with existing knowledge). This becomes computationally very hard when considering large metabolic models; however, a reduced application of GlobalFit that only looks at small subsets of experiments simultaneously works very well in practice. Allowing only changes that are conservative (e.g., introducing new reactions only if supported by significant genomic evidence), GlobalFit halves the number of wrong growth/non-growth predictions for the state-of-the-art metabolic models of *E*. *coli* and *Mycoplasma genitalium*, increasing prediction accuracy to 95.4% and 93.0%, respectively. By additionally allowing less conservative changes, we are able to improve accuracy further to 97.3% for the M. genitalium model.

## Introduction

Metabolism is the best understood large cellular system. Genome-scale metabolic models that largely rely on constraints for mass balance (i.e., all internal metabolites that are produced must also be consumed) are routinely applied to predict a wide range of metabolic phenomena [1]. The most widely-used of these constraint-based methods, Flux Balance Analysis (FBA), has been successfully applied to predict a range of biological phenomena such as gene knockout effects [1] and the evolutionary adaptation of microbial strains [2–4], and has been employed to predict drug targets [5] and to design microbial strains for bioengineering [6].

Network models are reconstructed by supplementing genomic annotation with information from biochemical characterizations and the organism-specific literature [7]. The resulting draft reconstructions often contain gaps: the modeled organism or its gene knockout strain can grow *in vivo*, while the model is unable to produce biomass *in silico* in the same metabolic environment (false-negative predictions, FNp). Gap filling methods have been introduced to resolve individual FNp through a minimal number of network changes, making irreversible reactions reversible or adding reactions from a database [8–11].

A second type of inconsistencies is the erroneous prediction of growth where the experiment shows no growth (false-positive predictions, FPp). Such cases can be rectified by deleting reactions, making reversible reactions unidirectional, or adding metabolites to the biomass (all reactions necessary for the production of a given metabolite become essential once this metabolite is added to the biomass). GrowMatch [12], the current state-of-the-art in automatic network refinement, uses bi-level optimization to identify reactions that must be deleted or modified for each FPp. GrowMatch also allows to add to the biomass products and/or substrates of reactions that are experimentally essential but are blocked in the model [12].

All currently available methods for network refinement based on growth data are greedy algorithms, solving one inconsistency between model and experiment at a time [8–15]. While each individual set of network changes is minimal, the union of these sets can become larger than a minimal set of changes that solves all inconsistencies simultaneously. Reactions considered essential or model changes introduced early may make the reconciliation of FNp or FPp considered later impossible (for an example, see our application to *Mycoplasma genitalium* below). Furthermore, experimental errors that happen to be consistent with the initial model can severely bias the results. Moreover, previous methods only alter the biomass equation independently of other network modifications [12, 16] and may miss solutions that combine biomass and network changes.

## Results

### An algorithm to find global rather than local optima when resolving inconsistencies

We present GlobalFit, a novel bi-level optimization method capable of comparing flux-balance analysis (FBA) [17] model predictions to measured growth across all tested environments and gene knockouts (or subsets thereof) simultaneously. Allowed model changes are (i) removals or (ii) reversibility changes of existing reactions; (iii) additions of reactions to the model from a database of potential reactions; (iv) removals of metabolites from the biomass; and (v) additions of metabolites to the biomass. GlobalFit does not change gene-protein-reaction associations (GPRs), and thus isoenzymes should be identified and included in the model as a preprocessing step.

The algorithm is first formulated as a bi-level linear problem, where each condition is represented by separate metabolites and fluxes (see the detailed method description in Methods). To ensure *in silico* growth for conditions with experimentally demonstrated growth, the biomass production for these conditions must be greater than a predefined threshold. For non-growth phenotypes, the inner optimization problem maximizes the biomass production to check whether it stays below a non-growth threshold. The outer optimization problem jointly minimizes the number of model changes and the number of experiments that are incorrectly predicted by the final model.

The penalties for individual network changes can be set independently. This allows, for example, to prefer reversibility changes over reaction additions, to preferentially remove reactions not associated with a gene, or to preferentially include additional reactions from metabolic network reconstructions of close relatives (see some suggestions for setting these penalties in the S1 Table). The bi-level problem can be re-formulated as a single-level optimization problem [18]; a corresponding implementation of GlobalFit, integrated with the *sybil* toolbox for constraint-based analyses [19], is freely available from CRAN (http://cran.r-project.org/web/packages/GlobalFit/).

While GlobalFit is designed to find globally optimal network modifications by considering all experimental data simultaneously, the corresponding MILP problem rapidly becomes prohibitively large when considering high-throughput gene knockout data. For example, simultaneously considering all possible 1366 *E*. *coli* knockouts [20] with 4000 allowed network modifications would result in a matrix with 13 million columns by 37 million rows, a problem size not addressable with current computing infrastructures.

However, when searching for model changes that rectify a FPp, trivial but unhelpful solutions such as the deletion of essential reactions are already avoided by simultaneously requiring growth in one or more specified true positive cases. When searching for model changes that rectify a FNp, overly generous changes (such as the removal of metabolites from the biomass) are avoided by simultaneously requiring non-growth in one or more specified true negative cases. Thus, while a globally optimal solution is only guaranteed when simultaneously considering all experimental growth data, a good approximation may be found by solving subsets of inconsistencies. We explore this “subset strategy” below in our application to the *E*. *coli* genome-scale model. We suggest contrasting each individual FPp with a wild-type growth case (or, if growth was assayed on different media, with a small set of wild-type growth cases). FNp may first be solved alone. However, if a suggested solution for a FNp or a FPp converts other previously correct predictions to false predictions (TPp to FNp or TNp to FPp), the originally considered case should be solved again, this time contrasting it with the complete set of these conflicting cases. This last step must be repeated until no more additional false predictions occur (or until no solution is found).

The runtime of MILP solvers depends crucially on the number of binary variables. Importantly, this number depends only on the number of allowed changes (plus a single binary variable for the inclusion/exclusion of each growth/non-growth case). Thus, a MILP strategy that considers *n* possible model changes for a single growth/non-growth case solves a problem with *n* binary variables. In comparison, the number of binary variables in a GlobalFit run that considers *n* possible model changes and contrasts *m* growth and non-growth cases is *n+m*. The number of binary variables can be further reduced by a set of preprocessing steps (Methods).

When reconciling a metabolic network with experimental data, the most parsimonious network modifications are not always those that best describe the true metabolic system. GlobalFit can also provide a specified number of alternative optimal or sub-optimal solutions (using the integer cut method). Thus, users can choose the solution(s) that best agree with available evidence, or design additional experiments that distinguish between competing network modifications. In cases where all suggested alternatives appear excessive or unrealistic, users may also consider modifying individual GPR rules. The runtime for *n* alternative solutions is approximately *n* times the runtime for a single optimum. In the test cases reported below, we only examined a small range of alternative solutions and did not consider manual modifications.

### Test case 1: Improving the iPS189 metabolic model for Mycoplasma genitalium

We first applied GlobalFit to the genome-scale metabolic network of *Mycoplasma genitalium* [21], using the same gene knockout essentiality data [22] as the initial reconstruction with GrowMatch (reported by [21] to have a global accuracy of 87.3%, corresponding to a Matthew’s correlation coefficient, a more balanced measure of classification quality [23], of MCC = 0.56; Table 1). The growth medium used for the knockout experiments was chemically undefined [22]. When applying GlobalFit, we thus allowed the uptake of all nutrients for which transport reactions are included in the model. All other FBA parameters were set to the values used in [21]. The initial network obtained from [21] was not able to produce biomass; to rectify this problem, we had to convert three irreversible reactions (*ZN2t4*,*INSK*,*LYSt3*) to reversible reactions. With these modifications, the original model [21] has an accuracy of 85% and a Matthews’ correlation coefficient MCC = 0.44. False predictions mainly occurred in the form of FPp, i.e., by incorrectly establishing growth *in silico* where a lethal phenotype was observed *in vivo* (Table 1).

**Table 1:** Comparison of experimental and predicted viability for 187 M. genitalium gene knockouts.

	Experiment			
Predictions	growth	non-growth	Accuracy	MCC
**GrowMatch** (reported in [21])^1^				
**growth**	16	22	87.3%	0.56
**no growth**	2	149		
**Unoptimized** model^2^				
**growth**	12	24	85.0%	0.44
**no growth**	4	147		
GlobalFit, conservative				
**growth**	14	10	93.6%	0.68
**no growth**	2	161		
GlobalFit, non-conservative				
**growth**	14	2	97.9%	0.86
**no growth**	2	169		

^1^ These numbers include the two genes wrongly associated with the FBA model (MG260, MG124) removed in our calculations.

^2^ The initial network obtained from [21] was not able to produce biomass in any environment; to rectify this problem, we converted three irreversible reactions (ZN2t4, INSK, LYSt3) to reversible reactions. We further allowed uptake of all metabolites for which transport reactions are included (see Methods).

To construct a database of potential additional reactions, we started from all reactions contained in metabolic networks provided by the BiGG database [24]. We removed globally blocked reactions, *i*.*e*., those reactions of the database that were not able to carry any flux in a supernetwork containing all reactions. Reversible reactions were represented as two independent irreversible reactions, corresponding to forward and backward directions. The database is provided as S2 Database of the supplementary material.

In our first analysis, we used a very restrictive, conservative set of potential network changes: (i) addition of reactions from other network reconstructions that are catalyzed by enzymes with significant sequence similarity to the *M*. *genitalium* genome (BLAST e-value <10^−13^); (ii) conversion of irreversible to reversible reactions for reactions that are at least classified as reversible with uncertainty in the *E*. coli model [25]; (iii) removal of reactions (separately for individual reaction directions for reversible reactions); (iv) removal of biomass components; and (v) addition of biomass components that occur in the biomass of other network reconstructions [16, 20, 24]. In this application, we assigned the same penalty (1.0) for all changes. However, as the growth medium used in the knockout experiments was undefined, we assigned a lower penalty (0.1) for the removal of exchange reactions. Thus, removal of a metabolite from the representation of the undefined medium (corresponding to the removal of an exchange reaction) was preferred to the removal of the corresponding transporter.

#### Solving false positive predictions (FPp)

14 out of 24 FPp could be transformed to true negatives (Tables 1 and 2), resulting in a specificity of 93.6%. Of the ten reactions that were suggested for removal, four were exchange reactions (for uracil, fructose, glycerol, and dATP), indicating the absence of these substrates from the undefined growth medium [22]; this alone solved a total of eight FPp. In each case, an alternative (though less parsimonious) solution would be the removal of the corresponding transport reaction (note, however, that the transport reactions for uracil and dATP have no associated gene).

**Table 2:** Modifications of the *M*. *genitalium* network suggested by GlobalFit based on 187 gene knockout experiments (bold font indicates conservative changes).

Type	Gene	Associated reactions	Removed reactions	Added reactions	Added biomass metabolite
FPp	***MG030***	UPPRT	NDPK1^for^, NDPK9^for^, URIK1^for^		
	***MG052***	CYTD, DCYTD	URAt2^for^ or EX_ura(e)		
	***MG053***	PMANM	PGAMT^back^ or G1PACT^for^	ACGAMPM^for^	
	***MG107***	DGK1, GK1, GK2	NDPK8^for^		
	***MG111***	G6PI,PGI	FRUpts^for^ or EX_fru(e)^back^		
	***MG187***	GLYC3Pabc	GLYCt^back^ or EX_glyc(e)^back^		
	***MG188***	GLYC3Pabc	GLYCt^back^ or EX_glyc(e)^back^		
	***MG189***	GLYC3Pabc	GLYCt^back^ or EX_glyc(e)^back^		
	***MG215***	PFK	FRUpts^for^ or EX_fru(e)^back^		
	***MG273***	PDH	DATPt^for^ or EX_datp(e)^back^		
	***MG274***	PDH	DATPt^for^ or EX_datp(e)^back^		
	***MG275***	NADH5	G3PD4^for^		
	***MG299***	PBUTT, PTA2r, PTAr	PGAMT^back^ or G1PACT^for^	ACGAMPM^for^	
	***MG357***	ACKr, PPAK	PGAMT^back^ or G1PACT^for^	ACGAMPM^for^	
	*MG038*	GLYK			Glycerol
	*MG050*	DRPAr			2-Deoxy-D-ribose 5-phosphate
	*MG137*	UDPGALM			UDP-D-galacto-1,4-furanose
	*MG259*	GLNMT			S-Adenosyl-L-homocysteine
	*MG356*	CHOLK		EX_chol(e), CHLabc^for^	Choline phosphate
	*MG372*	THZPSN			4-Hydroxy-benzyl alcohol and 4-Methyl-5-(2-phosphoethyl)-thiazole and 1-deoxy-D-xylulose 5-phosphate
	*MG396*	RPI			D-Ribulose 5-phosphate
	*MG448*	METSR-R1, METSR-R2			L methionine R oxide
*FNp*	***MG410***	PIabc		GLYK^back^	
	***MG411***	PIabc		GLYK^back^	

Four of the remaining six reactions indicated for removal (NDPK1, NDPK8, NDPK9, PGAMT) were not associated with a gene; i.e., they had an empty gene-protein-reaction association (GPR). A fifth reaction, G3PD4, is associated with the gene MG260; however, this association is likely erroneous. G3PD4 is catalyzed by a glycerol-3-phosphate dehydrogenase (1.1.5.3), whereas MG260 is a lipoprotein without significant sequence similarity to any proteins with known catalytic functions. Thus, GlobalFit suggests the removal of only one reaction (URIK1) that is reliably associated with a gene.

GlobalFit finds no network modification that predicts the lethality of MG124 knockouts. The gene MG124 encodes a thioreductase (THDPO) that is presumably used by Mycoplasma to protect itself from the consequences of self-generated oxidative challenges [26]. Its metabolic function is thus to regulate metabolite concentrations and cannot be captured in FBA models.

**Fig 1:**
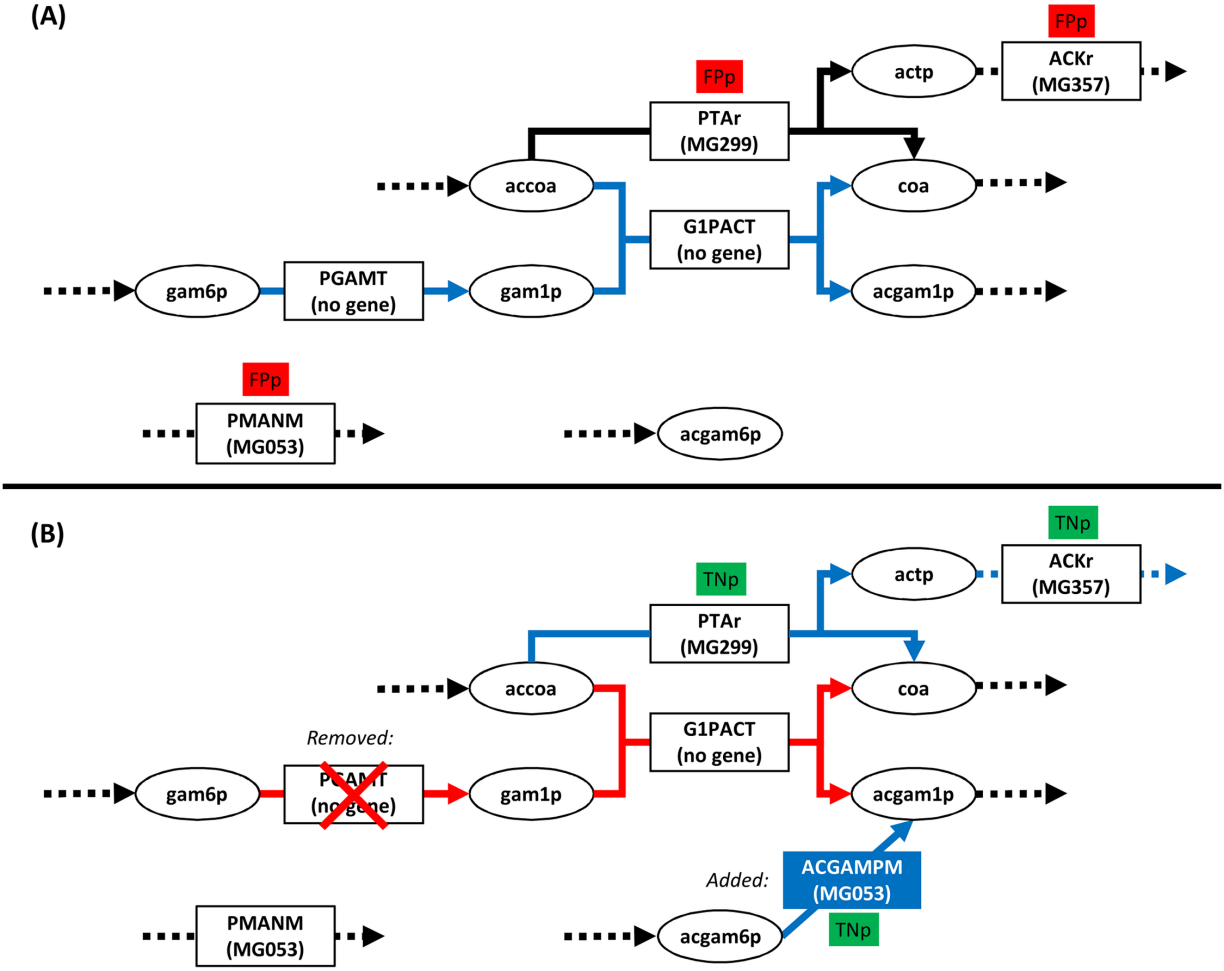
An example for the utility of simultaneously adding and removing reactions. Ellipses indicate metabolites, rectangles indicate reactions; abbreviations are taken from iPS189 [21]. (A) N-Acetyl-D-glucosamine 1-phosphate (acgam1p) is produced by G1PACT; MG053, MG299, and MG357 are falsely predicted to be non-essential (FPp). (B) The simultaneous removal of PGAMT (or, alternatively, G1PACT) and addition of ACGAMPM makes the genes MG053, MG299, and MG357 essential. Blue arrows mark essential pathways, while red arrows indicate blocked pathways. Note that removing either one of PGAMT or G1PACT blocks the other reaction, and that both reactions are not associated with any genes.

The remaining three solved FPp cases were corrected by simultaneously adding one reaction (ACGAMPM) and removing another (PGAMT). Without PGAMT, ACGAMPM is the only reaction producing N-Acetyl-D-glucosamine 1-phosphate, a precursor of the biomass metabolites teichuronic acid and minor teichoic acid (Fig 1). ACGAMPM is associated with three isoenzymes in the *M*. *tuberculosis* model [27], one of which shows strong sequence similarity to the *M*. *genitalium* genome. Notably, PGAMT is an essential reaction in the original network reconstruction [21], and would thus not be removed by previous algorithms that consider reaction additions and removals independently [12]. An alternative to the removal of PGAMT is the deletion of G1PACT; both reactions are not associated with any genes. G1PACT and PGAMT provide an alternative pathway to metabolize actetyl-CoA. Knocking out one of these genes, PTAr (MG299) and ACKr (MG357) become the only enzymes capable of metabolizing acetyl-CoA and thus become essential. Removing only G1PACT or PGAMT would seem to resolve the FPp for MG299 and MG357, but would result in a metabolic network unable to produce the essential biomass precursor N-Acetyl-D-glucosamine 1-phosphate and would thus be unviable.

Our second application of GlobalFit to the *M*. *genitalium* model followed [21] by allowing changes to all reactions and biomass metabolites. The resulting model changes form a superset of those proposed by the conservative analysis. We rectified FPp for 8 further cases, resulting in a specificity of 98.3%. All eight were resolved by adding metabolites to the biomass (Table 2); in one case, a further addition of two reactions was required (EX_chol(e), CHLabcfor; Table 2). Note that these biomass changes are not conservative; while they resolve inaccuracies *in silico*, they should be confirmed through further experiments. Previous studies [10, 12, 16] have also shown that modifying the biomass equation can improve the fit of model predictions to experimental growth data. However, estimating the correct biomass composition still remains a challenging task [7].

The two remaining unexplained FPp correspond to knocked-out genes associated with the same reaction as another gene whose knockout was a true positive prediction; thus, these predictions cannot be rectified without changing the gene-reaction associations.

The GlobalFit calculations for simultaneously solving all 11 feasible FPp cases (the number of unique enzyme complexes with FPp, Table 2) against the only FNp (two genes with FNp associated with the same reaction, Table 2) required 3h on a standard desktop computer (2 cores of an AMD Phenom 9600B 2.3GHz with 8GB RAM). However, as outlined above, the main advantage of GlobalFit is already played out when contrasting pairs of growth cases, which are much faster to solve. In the application to *M*. *genitalium*, we alternatively tested the subset strategy of first solving each FPp case separately against a wild-type control and each FNp alone; if the suggested solution turned the predictions for any other cases from true to false, we iteratively contrasted each case with the complete set of these negatively affected predictions. For the *M*. *genitalium* network, this approximate subset strategy resulted in the same proposed changes as the global analysis, while reducing the total computation time to below one minute. This result indicates that the application of GlobalFit is feasible even for very large growth datasets when employed in subset mode.

#### Solving false negative predictions (FNp)

FNp can be due to missing isoenzymes. Thus, an important pre-processing step to the application of GlobalFit is to identify homologous genes within the genome and to make corresponding changes to the GPRs. A blast e-value threshold of 10^−13^ has been used successfully before for isoenzyme identification in *E*. *coli* K12 [12]; however, we could not find any close homologs for the remaining two FNp mutants at this threshold.

For FNp, the results of the conservative and non-conservative application of GlobalFit were identical. Two FNp cases (Table 2), which together act as phosphate importers, could be resolved by allowing the reversibility of the phosphorylation of glycerol. This reaction is predicted to be reversible without uncertainty in *E*. *coli* [25]; furthermore, the glycerol kinase of *M*. *genitalium* shows strong sequence similarity (BLAST e-value 10^−136^) to the glycerol kinase of *Trypanosoma brucei*, which is known to indeed catalyze the reverse reaction [28, 29]. This single reversibility change increased sensitivity from 76.5% to 88.2%.

All modifications suggested by GlobalFit in the resolution of FPp and FNp cases were fully consistent with each other. In the highly conservative application of GlobalFit, we achieved an accuracy of 93.6% (MCC = 0.68; Table 1). If we follow previous work [21] by allowing all possible changes, GlobalFit obtains a global accuracy of 97.8%, and a Matthews correlation coefficient MCC = 0.86 (Table 1). The corresponding models differ only in their biomass reaction, and are supplied as S1 Model in SMBL format (non-conservative model: biomass reaction “Biomass”; conservative model: biomass reaction “Biomass_conservative”).

### Test case 2: Improving the iJO1366 metabolic model for *E*. *coli*

To test the applicability of GlobalFit’s subset strategy to larger models, we next applied it to the most recent genome-scale metabolic reconstruction for *E*. *coli*, iJO1366 [20]. Again, we employed the same gene knockout essentiality data [30, 31] as used in the initial reconstruction. For all FBA simulations, we used the same parameters as described in [20]. The maximal influx of all nutrients in the defined growth media was set to 10 mmol gDW^-1^h^-1^. The lower bound of the non-growth associated maintenance reaction (ATPM) was set to 3.15 mmol gDW^-1^h^-1^. Gene essentiality was then calculated by FBA, considering any flux larger than 5% of the optimal biomass core reaction as growth. For the published iJO1366 model, we obtained the same accuracies as reported originally [20]: a combined global accuracy of 90.8% calculated across knockout experiments on glucose and on glycerol media, corresponding to a Matthew’s correlation coefficient MCC = 0.67 (Table 3).

**Table 3:** Comparison of experimental and predicted viability for 1366 *E*. *coli* gene knockouts on two different minimal media.

	Experiment			
Predictions	growth	non-growth	Accuracy	MCC
Unoptimized model (iJO1366) grown on glucose				
growth	1079	80	91.3%	0.69
no growth	39	168		
Unoptimized model (iJO1366) grown on glycerol				
growth	1073	87	90.3%	0.66
no growth	45	161		
Optimized model grown on glucose				
growth	1104	45	95.7%	0.85
no growth	14	203		
Optimized model grown on glycerol				
growth	1096	44	95.2%	0.83
no growth	22	204		

In the application of GlobalFit to the iJO1366 model, we only allowed conservative network modifications (as defined for the *M*. *genitalium* model). However, as the growth medium used in the *E*. *coli* experiments was chemically defined, we did not allow the removal of exchange reactions. We constructed a database of potential new reactions as for *M*. *genitalium* (S2 Database).

The knockout data for *E*. *coli* includes growth data on two different media that contained either glucose or glycerol as carbon sources [30, 31]. Accordingly, we solved all FPp against two wild-type growth cases, one on glucose and one on glycerol. While this increases the number of continuous variables compared to using only a single wild-type growth case, the number of binary variables is still the same as in algorithms that only consider a single non-growth case at a time [12] (note that we don’t allow the exclusion of any growth/non-growth case in this application). We tested if the order in which false growth/non-growth predictions are considered in GlobalFit’s subset strategy affects the final result; this was not the case.

By applying the network modifications suggested by GlobalFit, we could strongly increase the quality of predictions for growth on both glycerol and glucose (Table 3); for the experiments on glucose and on glycerol combined, accuracy increased from 90.8% to 95.4%, while Matthew’s correlation coefficient increased from 0.67 to 0.84. The detailed model changes are outlined below.

#### Solving FNp: Isoenzymes

One simple explanation for FNp is the existence of un-annotated isoenzymes. To detect such cases, we identified all FNp where the knocked-out gene has a significant bi-directional blast hit with another gene in the genome (*i*.*e*., BLAST e-value < 10^−13^ for the other gene when using either of the two as query). Such highly conserved homologs are likely to be functionally very similar to the knocked-out gene [12], and we updated the GPR accordingly. We only performed this analysis for those genes that were reported to be non-essential on both glucose and glycerol. In this way, we could convert six FNp to TPp (Table 4). In two cases (b0888 and b1702), the requirement for the inclusion of isoenzymes was not previously recognized, as the iJO1366 model wrongly included an alternative pathway; solving a FPp related to the alternative pathway converted the TPp into a FNp that was then rescued by the inclusion of the newly identified isoenzymes.

**Table 4:** Isoenzymes that resolved FNp.

Gene	Associated reactions	Isoenzyme	e-value →	e-value ←
*b0888*	TRDR	b0606	2x10^-35^	8x10^-37^
*b0928*	ASPTA	b4054	2x10^.113^	2x10^-113^
*b1415*	GCALDD, LCADi	b1385	7x10^-80^	1x10^-77^
*b1702*	PPS	b2383	2x10^-22^	2x10^-22^
*b3176*	PGAMT	b2048	3x10^-16^	1x10^-18^
*b3359*	SDPTA	b1748	1x10^-180^	1x10^-180^

#### Solving FNp: Removing biomass components

Removing metabolites from the biomass reaction can convert FNp to TPp, as all genes involved in the production (or, if the metabolite was a product of the biomass reaction, consumption) of a metabolite become unessential. GlobalFit suggested the removal of six metabolites from the biomass reaction, thereby resolving 19 FNp (Table 5). For example, removing Bis-molybdopterin guanine dinucleotide from the biomass reaction converted eight genes involved in the synthesis of this metabolite from essential to non-essential genes. By removing Bis-molybdopterin guanine dinucleotide and Thiamine diphosphate, two TNp become FPp (b0417 and b2530); however, because these two changes also correct 16 FNp, the overall accuracy was strongly increased.

**Table 5:** Removal of biomass components from the *E*. *coli* model suggested by GlobalFit to remove FNp.

Gene	Associated reactions	Removed biomass metabolite
*b0009*	MPTAT	Bis-molybdopterin guanine dinucleotide
*b0423*	THZPSN3	Thiamine diphosphate
*b0781*	CPMPS	Bis-molybdopterin guanine dinucletide
*b0783*	CPMPS	Bis-molybdopterin guanine dinucletide
*b0784*	MOADSUx, MPTS	Bis-molybdopterin guanine dinucletide
*b0785*	MPTS	Bis-molybdopterin guanine dinucletide
*b0826*	MPTSS	Bis-molybdopterin guanine dinucletide
*b0827*	BMOCOS, BWCOS, MOCOS, WCOS	Bis-molybdopterin guanine dinucletide
*b2103*	PMPK	Thiamine diphosphate
*b3040*	CD2tpp, CU2tpp, FE2tpp, MN2tpp, ZN2tpp	Copper
*b3196*	CAt6pp	Calcium
*b3807*	I2FE2SS, I2FE2SS2, S2FE2SS, S2FE2SS2	[4Fe-4S] iron-sulfur cluster and [2Fe-2S] iron-sulfur cluster
*b3857*	BMOGDS1, BMOGDS2, BWCOGDS1, BWCOGDS2, MOGDS	Bis-molybdopterin guanine dinucletide
*b3990*	THZPSN3	Thiamine diphosphate
*b3991*	TYRL	Thiamine diphosphate
*b3992*	THZPSN3	Thiamine diphosphate
*b3993*	TMPPP	Thiamine diphosphate
*b3994*	AMPMS2	Thiamine diphosphate
*b4407*	THZPSN3	Thiamine diphosphate

GlobalFit further indicated the removal of calcium and copper from the biomass, which was also suggested by the BioMog algorithm based on *E*. *coli* growth data on different media [16]. Calcium is essential for proper functioning of *E*. *coli* chemotaxis [32]. However, compromised chemotaxis will not be detected in the knockout experiments. Thus, we suggest to retain calcium in the biomass reaction when modeling *E*. *coli* in its natural habitat, but to remove calcium from the biomass reaction when modeling *E*. *coli* in cell culture.

#### Solving FNp: Reversing reactions

Five FNp could be resolved by reversing existing reactions in the metabolic network (Table 6). Interestingly, an alternative solution for two genes was to remove calcium or copper from the biomass reaction. For calcium, the above arguments indicate that its removal from the biomass reaction may be preferable.

**Table 6:** Reversal of reactions of the *E*. *coli* network suggested by GlobalFit to remove FNp.

Gene	Associated reactions	Reversed reactions
*b0159*	5DOAN, AHCYSNS, MTAN	HCYSMT, CPPPGO2
*b2103*	PMPK	2MAHMP
*b2687*	RHCCE	HCYSMT
*b3040*	CD2tpp, CU2tpp, FE2tpp, MN2tpp, ZN2tpp	CU2abcpp
*b3196*	CAt6pp	CA2t3pp

#### Solving FNp: Adding new reactions to the network

GlobalFit could not improve the accuracy of knockout predictions by adding new reactions to the metabolic network. This may have several reasons. First, the reconstruction of the *E*. *coli* metabolic network iJO1366 involved extensive literature and database searches to ensure a maximal inclusion of metabolic reactions. Second, we used the BiGG database as the source for potential additional reactions. Many networks in this database are based on the *E*. *coli* network reconstruction; this makes it unlikely that they provide new features relevant for *E*. *coli*. Third, the cut-off value for the similarity of enzymes to the *E*. *coli* genome used in the construction of the additional reaction database might have been too strict (10^−13^).

#### Solving FPp: Adding metabolites to the biomass reaction

22 FPp could be resolved by adding metabolites as substrate or product to the biomass reaction (Table 7). 17 of these corresponded to (previously blocked) tRNA charging reactions; these were resolved by adding charged and uncharged tRNA metabolites to the two sides of the biomass reaction, respectively, similar to previous suggestions for the older iAF1260 *E*. *coli* model [12]. GrowMatch only considers additions to the biomass if a gene with a FPp catalyzes a blocked reaction; it then tests the addition of the metabolites consumed or produced by this reaction [12]. However, none of the genes for the remaining five FPp resolved by GlobalFit through biomass additions catalyzed blocked reactions. When allowing the addition of biomass components not included in other BiGG biomass reactions or suggested by BioMog, GlobalFit was able to resolve 4 additional FPp (for b2533, b2925, b3623, b3650); however, as these suggested modifications did not meet our strict criteria, we did not consider them further.

**Table 7:** Metabolite additions to the *E*. *coli* biomass reaction suggested by GlobalFit to resolve FPp.

Gene	Associated reactions	Added as biomass substrate	Added as biomass product
*b0194*	PROTRS	L-Prolyl-tRNA(Pro)	TRNA(Pro)
*b0242*	GLU5K	L-Glutamate 5-phosphate	
*b0526*	CYSTRS	L-Cysteinyl-tRNA(Cys)	TRNA(Cys)
*b0529*	MTHFC, MTHFD	5-Formyltetrahydrofolate	
*b0642*	LEUTRS	L-Leucyl-tRNA(Leu)	TRNA(Leu)
*b0680*	GLNTRS	L-Glutaminyl-tRNA(Gln)	TRNA(Gln)
*b0893*	SERTRS, SERTRS2	L-Seryl-tRNA(Ser)	TRNA(Ser)
*b0930*	ASNTRS	L-Asparaginyl-tRNA(Asn)	TRNA(Asn)
*b1637*	TYRTRS	L-Tyrosyl-tRNA(Tyr)	TRNA(Tyr)
*b1713*	PHETRS	L-Phenylalanyl-tRNA(Phe)	TRNA(Phe)
*b1714*	PHETRS	L-Phenylalanyl-tRNA(Phe)	TRNA(Phe)
*b1719*	THRTRS	L-Threonyl-tRNA(Thr)	TRNA(Thr)
*b1866*	ASPTRS	L-Aspartyl-tRNA(Asp)	TRNA(ASP)
*b1876*	ARGTRS	L-Arginyl-tRNA(Arg)	TRNA(ARG)
*b1912*	PGSA120, PGSA140, PGSA141, PGSA160, PGSA161, PGSA180, PGSA181	Phosphatidylglycerophosphate (didodecanoyl, n-C12:0) or Phosphatidylglycerophosphate (ditetradecanoyl, n-C14:0) or Phosphatidylglycerophosphate (ditetradec-7-enoyl, n-C14:1) or Phosphatidylglycerophosphate (dihexadecanoyl, n-C16:0) or Phosphatidylglycerophosphate (dihexadec-9-enoyl, n-C16:1) or Phosphatidylglycerophosphate (dioctadecanoyl, n-C18:0) or Phosphatidylglycerophosphate (dioctadec-11-enoyl, n-C18:1)	
*b2114*	METTRS		TRNA(Met)
*b2514*	HISTRS	L-Histidyl-tRNA(His)	TRNA(His)
*b2551*	GHMT2r, THFAT	5-Formyltetrahydrofolate	
*b2913*	PGCD	3-Phosphohydroxypyruvate	
b3288	FMETTRS	N-Formylmethionyl-tRNA	
b3384	TRPTRS	L-Tryptophanyl-tRNA(Trp)	TRNA(Trp)
b4258	VALTRS	L-Valyl-tRNA(Val)	TRNA(Val)

#### Solving FPp: Removing reactions

25 FPp could be resolved by removing a total of 18 reactions from the metabolic network (Table 8). At the same time, four TPp were converted to FNp; however, two of these newly introduced FNp could subsequently be corrected through additional network modifications.

**Table 8:** Removal of reactions of the *E*. *coli* network suggested by GlobalFit to correct FPp.

Gene	Associated reactions	Removed reactions
*b0032*	CBPS	(CBMKr^for^ and ALLTAMH^for^) or (CBMKr^for^ and ALLTN^for^) or (CBMKr^for^ and OXAMTC^for^) or (CBMKr^for^ and URDGLYCD^for^) or (CBMKr^for^ and URIC^for^)
*b0033*	CBPS	(CBMKr^for^ and ALLTAMH^for^) or (CBMKr^for^ and ALLTN^for^) or (CBMKr^for^ and OXAMTC^for^) or (CBMKr^for^ and URDGLYCD^for^) or (CBMKr^for^ and URIC^for^)
*b0242*	GLU5K	NACODA^for^
*b0243*	G5SD	NACODA^for^
*b0474*	ADK1, ADK3, ADK4, ADNK1, DADK	NDPK1^for^ or PRPPS^back^ or R1PK^for^ or PPM^back^ or R15BPK^for^
*b0945*	DHORD2, DHORD5	DHORDfum^for^
*b0954*	T2DECAI	(CTECOAI6^back^ and CTRCOAI7^back^) or (CTECOAI6^back^ and AACPS4^for^)
*b1207*	PRPPS	R1PK^for^ or PPM^back^ or R15BPK^for^
*b1638*	PDX5POi, PYAM5PO	PDX5PO2^for^
*b1779*	GAPD	TPI^for^
*b2234*	RNDR1, RNDR2, RNDR3, RNDR4	(GRXR^for^ and RNTR3c2^for^) or (GTHOr^for^ and RNTR3c2^for^) or (GRXR^for^ and RNTR1c2^for^) or (GTHOr^for^ and RNTR1c2^for^)
*b2235*	RNDR1, RNDR2, RNDR3, RNDR4	(GRXR^for^ and RNTR3c2^for^) or (GTHOr^for^ and RNTR3c2^for^) or (GRXR^for^ and RNTR1c2^for^) or (GTHOr^for^ and RNTR1c2^for^)
*b2508*	IMPD	HXAND or XPPT
*b2913*	PGCD	GHMT2r^back^
*b2926*	PGK	TPI^for^
*b3731*	ATPS4rpp	(F6PA^back^ and PGK^back^) or (G6PDH2r^for^ and PGK^back^)
*b3733*	ATPS4rpp	(F6PA^back^ and PGK^back^) or (G6PDH2r^for^ and PGK^back^)
*b3734*	ATPS4rpp	(F6PA^back^ and PGK^back^) or (G6PDH2r^for^ and PGK^back^)
*b3735*	ATPS4rpp	(F6PA^back^ and PGK^back^) or (G6PDH2r^for^ and PGK^back^)
*b3736*	ATPS4rpp	(F6PA^back^ and PGK^back^) or (G6PDH2r^for^ and PGK^back^)
*b3738*	ATPS4rpp	(F6PA^back^ and PGK^back^) or (G6PDH2r^for^ and PGK^back^)
*b3835*	OPHHX	OPHHX3^for^
*b3956*	PPC	FUM^for^ or MALS^for^
*b4041*	G3PAT120, G3PAT140, G3PAT141, G3PAT160, G3PAT161, G3PAT180, G3PAT181	ACPPAT160^for^ or AG3PAT161^for^ or AG3PAT160^for^
*b4388*	PSP_L	GHMT2r^back^

One example is the ATP synthase reaction ATPS4rpp, which is catalyzed by an enzyme complex encoded by eight genes. When *E*. *coli* was grown on glycerol, six of these genes were essential, while on glucose only three genes were found to be essential. Thus, overall accuracy is optimized if ATPS4rpp is essential for growth on glycerol, but non-essential for growth on glucose. We used GlobalFit to simultaneously solve a non-growth case of the ATPS4rpp knockout on glycerol, a wild-type growth case on glycerol, and a growth case of the ATPS4rpp knockout on glucose. GlobalFit found two alternative solutions that make the Phosphoglycerate kinase reaction irreversible (removing the backward direction of PGK) and also make the Fructose 6-phosphate aldolase reaction (F6PA^back^) or the Glucose 6-phosphate dehydrogenase (G6PDH2r^for^) irreversible. By applying either of these two modifications, the two TPp of ATP synthase subunits for glycerol were converted to FNp.

For two of the 25 solved FPp (b0242 and b2913), alternative solutions are provided by adding metabolites to the biomass reaction (Table 7). For example, the FPp of b2913 (encoding Phosphoglycerate dehydrogenase) could be resolved by making the Glycine hydroxymethyltransferase reaction (GHMT2r) irreversible. An alternative solution is the addition of 3-Phosphohydroxypyruvate (3php) to the biomass reaction, which was also suggested by BioMog [16]. However, only the removal of GHMT2r simultaneously resolved the FPp of b4388 (Phosphoserine phosphatase (L-serine)).

#### Solving FPp: Other

On glucose, 19 of the remaining 45 FPp corresponded to isoenzymes; on glycerol, 21 of the 33 remaining FPp corresponded to isoenzymes. FBA models do not account for gene regulation, and thus the corresponding reactions are assumed to remain active even when knocking out one of the isoenzymes. Thus, these FPp are due either to erroneous GPRs or to the isoenzymes not being expressed. GlobalFit does not allow changes to GPRs or inclusion of regulatory rules, and, consequently, could not find any solution for these genes.

The resulting modified model of *E*. *coli* metabolism is provided as S2 Model in SBML format.

## Discussion

In this work, we describe and implement a novel algorithm to automatically modify metabolic network models based on growth/non-growth data. The algorithm can utilize data from different growth environments and/or different gene knockouts. In contrast to previous approaches, the “global” mode of GlobalFit does not reconcile the network model with inconsistent experiments iteratively, but finds a globally minimal set of network changes that resolves all inconsistencies simultaneously (in so far as the inconsistencies are resolvable with the allowed model modifications). To make GlobalFit applicable to large metabolic network reconstructions, we also explored a subset strategy, where individual false predictions are solved simultaneously with small subsets of growth/non-growth cases.

We demonstrate the utility of these approaches through applications to the previously published network models of *M*. *genitalium* [21] (optimizing model predictions for gene knockout data from Ref. [22]) and *E*. *coli* [20] (utilizing gene knockout data from Ref. [30, 31]). Allowing only highly conservative network changes (e.g., only adding reactions catalyzed by enzymes that are homologous to genes of the species studied), we were able to halve the number of false growth predictions in each case. Overall, GlobalFit improved the accuracy of growth/non-growth predictions for *M*. *genitalium* from 87.3% to 93.6% (MCC from 0.56 to 0.68) and for *E*. *coli* from 90.8% to 95.4% (MCC from 0.67 to 0.84). If we allow a much wider range of possible network modifications—which is routinely done in alternative approaches [12, 21]–even higher accuracies can be achieved. Importantly, GlobalFit can enumerate alternative optimal or sub-optimal solutions, such that expert knowledge or additional experiments can help select the biologically most realistic modifications.

For some inconsistencies, we found solutions that improved accuracy on one medium while decreasing accuracy on the other. For example, adding selenium to the biomass reaction of *E*. *coli* would resolve three FPp on glycerol, while converting four TPp to FNp on glucose. Thus, the accuracy achievable for one growth medium could be further improved by sacrificing the accuracy for the other medium, albeit at a likely loss of biological correctness. This observation emphasizes the utility of combining gene knockout data across different nutritional environments to avoid problems of overfitting.

In other cases, several genes whose products act together in a protein complex had contradictory experimental results: in the same medium, some were found to be essential, while the rest was declared non-essential. Such contradictions may be caused either by experimental errors, by erroneous assignment of genes to reactions (incorrect GPRs), or by a residual function of the enzyme complex even with some of its components missing. GlobalFit may suggest a solution in this case, but this will simultaneously distort one or more true predictions. For example, the FPp for the *E*. *coli* gene b3560 (the α-subunit of glycine tRNA synthetase) could be resolved by adding the charged and uncharged glycine tRNA to the biomass reaction as substrate and product, respectively. This modification would at the same time transform the TPp of b3559 (the β-subunit) to a FNp, and would thus not improve accuracy.

In the applications of GlobalFit, we adopted the *in silico* growth cutoffs used in the original model publications, *i*.*e*., one third of the mean growth rate for *M*. *genitalium* [21] and 5% of the optimal biomass core reaction for *E*. *coli* [20]. A more general way to resolve FPp would be to treat the cutoff that distinguishes *in silico* growth from non-growth as an additional variable in the optimizations. For example, the knockout of *E*. *coli* ATPS4rpp reduced the biomass yield in glycerol below 10% of the wild-type yield. Such a substantial reduction in growth rate may explain why 6 out of 8 knockouts for the genes involved in the corresponding enzyme complex were labeled as essential in the experiment; however, following [20] in considering 5% biomass production as growth, we regarded these knockouts as FPp in this study. An adjustable growth threshold might have rectified these FPp cases without any model changes. It is not clear *a priori* which *in silico* cutoff corresponds best to a given set of experimental data. Thus identifying the cutoff value that minimizes the necessary model changes seems most appropriate.

In this paper, we have explored the application of GlobalFit to the improvement of existing metabolic network reconstructions and showed that it can substantially reduce the number of false growth predictions even when restricted to conservative network changes. It is conceivable that GlobalFit can also be employed for other tasks related to metabolic model refinement. One possible such application is the initial reconstruction of a metabolic network model starting from a computer-generated template that is based on genome annotation (such as provided, e.g., by the SEED algorithm [33]). GlobalFit might also be used to remove thermodynamically impossible energy-creating cycles, which sometimes plague initial network reconstructions. While we only score growth and non-growth, GlobalFit could also be applied using yield data by choosing appropriate thresholds. Finally, we envisage future usage of *GlobaFit* for strain optimization in metabolic engineering applications that combine gene knockouts [34] with gene additions.

## Methods

### Formal problem definition

GlobalFit compares flux-balance analysis (FBA) [17] model predictions to measured growth across all tested environments and gene knockouts simultaneously. Allowed model changes are (i) removals or (ii) reversibility changes of existing reactions; (iii) additions of reactions to the model from a database of potential reactions; (iv) removals of metabolites from the biomass; and (v) additions of metabolites to the biomass.

We thus solve the following bi-level problem:

$${\text{min}}_{\underset{\delta }{\to }}\left(\sum_{y\in M}\left(\delta_{y}^{RF}+\delta_{y}^{RB}\right)\times w_{y}^{R}+\sum_{x\in I}\delta_{x}^{I}\times w_{x}^{I}+\sum_{z\in D}\delta_{z}^{add}\times w_{z}^{add}+\sum_{j\in A_{S}}\delta_{j}^{AS}\times w_{j}^{AS}+\sum_{k\in A_{P}}\delta_{k}^{AP}\times w_{k}^{AP}+\sum_{l\in B_{S}}\delta_{l}^{RS}\times w_{l}^{RS}+\sum_{m\in A_{P}}\delta_{m}^{RP}\times w_{m}^{RP}+\sum_{g\in G}\delta_{g}^{G}\times w_{g}^{G}+\sum_{h\in N}\delta_{h}^{N}\times w_{h}^{N}\right)$$ (1)

*subject to*:

$$\forall_{g\in G}S\times v_{g}=0$$ (2)

$$\forall_{h\in G}S\times v_{h}=0$$ (3)

$$\forall_{y\in M,\;g\in G\cup N}\;v_{y}^{min}\times \left(1-\delta_{y}^{RB}\right)\leq \;v_{y}^{g}\leq \;v_{y}^{max}\times \left(1-\delta_{y}^{RF}\right)$$ (4)

$$\forall_{x\in I,\;g\in G\cup N}-1000\times \delta_{x}^{I}\leq v_{x}^{g}$$ (5)

$$\forall_{z\in D,\;\;\;g\in G\cup N}\;0\leq \;v_{z}^{g}\leq 1000\times \delta_{z}^{add}$$ (6)

$$\forall_{y\in M,\;\;\;g\in G\cup N}\sum {}_{l\in B_{S}}(1-\delta_{l}^{RS})\times c_{l}^{RS}+\sum {}_{j\in A_{S}}\delta_{j}^{AS}\times c_{j}^{AS}\overset{v_{Bio}^{g}}{\to }\sum {}_{m\in B_{P}}(1-\delta_{m}^{RP})\times c_{m}^{RP}+\sum {}_{k\in A_{P}}\delta_{k}^{AP}\times c_{k}^{AP}$$ (7)

$$\forall_{g\in G}\;\left(v_{Bio}^{g}+1000\times \;\delta_{Bio}^{iG}\geq \;T_{g}\right)$$ (8)

$$\forall_{h\in N}\;\left({\hat{v}}_{Bio}^{h}-1000\times \;\delta_{Bio}^{iN}\leq \;T_{h}\right)$$ (9)

*with*:

$$Inner\;Problem:{\hat{v}}_{Bio}^{h}∶=\;\underset{{\vec{v}}^{h}}{\text{max}}v_{Bio}^{h},$$ (10)

*subject to*: Eqs (3)–(7) *and to the definitions following below*.

Line (7) defines the flux through the biomass reaction, $v_{Bio}^{g}$, for condition *g*. The sets used in this system of equations are listed in Table 9, while the parameters are defined in Table 10. For binary variables, 1 corresponds to TRUE (i.e., a model change is executed), while 0 corresponds to FALSE (no change compared to the initial network).

**Table 9:** Definitions of the sets used in the system of equations that describes the GlobalFit algorithm.

*M*	The reactions included in the original (input) network reconstruction
*I*	All irreversible reactions that can be reversed
*D*	All reactions that can be added to the network (here, we consider bidirectional reactions as two separate reactions corresponding to forward and backward directions (with fluxes ≥0)).
B_S_	All substrates that can be removed from the biomass reaction
*c*^*BS*^	The stoichiometric coefficients of all biomass substrates
B_P_	All products that can be removed from the biomass reaction
*c*^*BP*^	The stoichiometric coefficients of all biomass products
*A*_*S*_	All substrates that can be added to the biomass reaction
*c*^*AS*^	The stoichiometric coefficients of all additional biomass substrates
*A*_*P*_	All products that can be added to the biomass reaction
*c*^*AP*^	The stoichiometric coefficients of all additional biomass products
*G*	All experiments with observed growth
*N*	All experiments with observed non-growth

**Table 10:** The parameters of the system of equations describing the GlobalFit algorithm.

$\delta_{y}^{RF},\;\delta_{y}^{RB}\in \;\left\{0,1\right\}$	Binary variables that indicate the removal of forward and backward reaction *y*, respectively
$w_{y}^{R}>0$	Penalty for the removal of forward or backward reaction (which can be set to a different value for each reaction *y*)
$\delta_{x}^{I}\in \;\left\{0,1\right\}$	Binary variables that indicate the addition of a backward reaction for reaction *x*
$w_{x}^{I}>0$	Corresponding penalties
$\delta_{z}^{add}\in \;\left\{0,1\right\}$	Binary variables that indicate the addition of reaction *z*
$w_{z}^{add}>0$	Corresponding penalties
$\delta_{j}^{AS}\in \left\{0,1\right\}$	Binary variables that indicate the addition of substrate *j* to the biomass reaction
$w_{j}^{AS}>0$	Corresponding penalties
$\delta_{k}^{AP}\in \;\left\{0,1\right\}$	Binary variables that indicate the addition of product *k* to the biomass reaction
$w_{k}^{AP}>0$	Corresponding penalties
$\delta_{l}^{RS}\in \;\left\{0,1\right\}$	Binary variables that indicate the removal of substrate *l* from the biomass reaction
$w_{l}^{RS}>0$	Corresponding penalties
$\delta_{m}^{RP}\in \;\left\{0,1\right\}$	Binary variables that indicate the removal of product *m* from the biomass reaction
$w_{m}^{RP}>0$	Corresponding penalties
$\delta_{g}^{G}\in \;\left\{0,1\right\}$	Binary variables that indicate the exclusion of growth experiment *g*
$w_{g}^{G}>0$	Corresponding penalties
$\delta_{h}^{N}\in \;\left\{0,1\right\}$	Binary variables that indicate the exclusion of non-growth experiment *h*
$w_{h}^{N}>0$	Corresponding penalties
$\;v_{Bio}^{g}$	Flux through the (potentially modified) biomass reaction (see line (7))
$\;{\hat{v}}_{Bio}^{g}$	Optimal value for $\;v_{Bio}^{g}$ estimated in the inner problem
$\;v_{y}^{min}\leq 0$	Minimal flux allowed through reaction *y* (note that we do not allow minimal fluxes >0 for non-growth cases)
$\;v_{y}^{max}\geq 0$	Maximal flux allowed through reaction *y* (note that we do not allow maximal fluxes <0 for non-growth cases)
$T_{g}>0$	Viability threshold of growth experiment *g*
$T_{h}>0$	Viability threshold of non-growth experiment *h*
$\vec{\delta }$	The vector of all *δ* defined above
${\vec{v}}^{h}$	The vector of all fluxes $\;v_{i}^{h}$ for experiment *h*

### GlobalFit’s logic

What is the purpose of each of the lines in the above system of equations? The network must be in a steady state (*i*.*e*., no concentration changes to internal metabolites) in all conditions *g* ∈ *G* Eq (2) and *h* ∈ *N* Eq (3) that are to be solved simultaneously.

Lines (4)–(6) convert the binary variables for the removal or reversibility change of existing reactions, and for the addition of new reactions from the database, into constraints for the respective fluxes. In Eq (4), if $\delta_{y}^{RB}=0$ (*i*.*e*., no change), then the lower limit for reaction *y* in all conditions *g* ($v_{y}^{g}$) remains at the predefined limit $v_{y}^{min}$; setting $\delta_{y}^{RB}=1$ instead sets the lower flux limit to 0, *i*.*e*., removes the backwards reaction. Similarly, setting $\delta_{y}^{RF}=0$ keeps the upper flux limit for reaction *y* at the predefined limit $v_{y}^{max}$, while setting $\delta_{y}^{RF}=1$ sets the upper flux limit to 0, *i*.*e*., removes the forward reaction.

Line (5) sets the lower flux limit to -1000 for reaction *y* in all conditions *g* if $\delta_{x}^{I}=1$, i.e., it makes an irreversible reaction (with flux $v_{x}^{g}\geq 0$) reversible in this case. Line (6) allows non-zero (positive) flux for reactions that are not part of the original (input) model if $\delta_{z}^{add}=1$. Note that in the database of additional potential reactions, we consider bidirectional reactions as two separate reactions corresponding to forward and backward directions (both with fluxes ≥0).

Metabolites can be removed from both sides of the biomass reaction (flux $v_{Bio}^{g}$), and additional metabolites can be added Eq (7) with pre-specified stoichiometric coefficients *c*.

To ensure *in silico* growth for conditions with experimentally demonstrated growth, the biomass flux for these conditions must be greater than a predefined threshold *T*_*g*_ in all conditions *g* ∈ *G* Eq (8). Conversely, to ensure *in silico* non-growth for conditions with experimentally demonstrated non-growth, the biomass flux for these condition must be less than a predefined threshold *T*_*h*_ in all conditions *h* ∈ *N* Eq (9). The thresholds *T*_*g*_ and *T*_*h*_ can be set separately for each phenotype, *e*.*g*., to account for estimates of experimental errors. For non-growth phenotypes, a simple condition that forces the biomass production to be lower than a threshold is not sufficient, though, as a trivial solution with ${\vec{v}}^{h}=0$ would satisfy this condition. To overcome this problem, the inner optimization problem maximizes the biomass production of non-growth cases Eq (9), and this maximum is compared against the non-growth threshold.

Line (1) describes the outer optimization problem. GlobalFit aims to find a solution that is able to correctly predict all growth and non-growth cases with a minimal number of network changes (indicated by values 1 for the binary variables):

$$\delta_{y}^{RF},\;\delta_{y}^{RB},\;\delta_{x}^{I},\;\delta_{z}^{add},\delta_{j}^{AS},\;\delta_{k}^{AP},\;\delta_{l}^{RS},\;\delta_{m}^{RP},\;\delta_{g}^{G},\delta_{h}^{N}$$

The penalties for each type of network change, and even for each individual change, can be set independently. This allows, for example, to prefer reversibility changes over reaction additions, or to preferentially include new reactions with stronger genomic evidence, or reactions from metabolic network reconstructions of close relatives. Users should choose appropriate penalties based on the details of the network reconstruction and the proposed changes. As a starting point, we include a list of suggested penalty values in S1 Table).

To guarantee a feasible solution, even if inconsistent growth cases are used, we implemented additional binary variables that allow the exclusion of individual growth ($\delta_{g}^{G}$ Eq (8)) and non-growth cases ($\delta_{h}^{N}$ Eq (9)) from the growth threshold conditions. In our application to the *M*. *genitalium* network, we penalize these condition exclusions with very high values $w_{g}^{G}$ and $w_{h}^{N}$; thus, any network modification that explains additional cases is preferred over the exclusion of conditions, regardless of the number of required changes. Instead, the penalties can be set to smaller values, so that the exclusion of potentially erroneous experiments is preferred over excessive network changes.

Metabolic network reconciliation with large-scale experimental data usually incorporates a manual curation stage, where experts for the physiology and biochemistry of the organism under study review network changes suggested by automated methods. To support this process, GlobalFit can put out not just one best solution, but, *e*.*g*., the five best solutions that can then be reviewed to identify the changes most compatible with existing knowledge. To speed up the calculations, network changes can also be limited to a maximal number.

### Re-formulation of the bi-level as a single level optimization problem

No efficient software tools for general bi-level optimization problems are available. Solving the inner problem for each possible combination of network changes would be computationally too slow. We adapt the “Reduction Ansatz” of Section 4.3.4 in [18] to eliminate the inner problem in line (9). In this approach, the optimality conditions of the inner optimization problem are expressed as equality and inequality conditions using additional “dual” variables. For fixed $\vec{\delta }$ and *h*, the inner problem is simply a linear program; thus, the assumptions in [18] are trivially satisfied.

Because of the use of binary variables, algorithms to solve this type of optimization problem are termed mixed integer linear programming (MILP). MILP is NP hard [35]; while no known algorithms can guarantee to find a solution efficiently, algorithms that work well for many practical problems exist in software solvers. We used the solver of IBM ILOG CPLEX 12.5; to avoid trickle flow, we implemented indicator constraints. Alternatively, our implementation of GlobalFit also allows using the GUROBI solver. Academic users can obtain both CPLEX and GUROBI free of charge.

### Preprocessing

The search for a globally minimal set of network changes is a computationally very intensive task. To speed up this process, it is advisable to restrict the examined conditions to a maximal consistent (“feasible”) set, *i*.*e*., a maximal set of conditions that can all be correctly predicted with the same modified metabolic network (regardless of the type and number of modifications). To identify such feasible condition sets, GlobalFit provides a *simple mode*, which only minimizes the number of erroneous predictions of growth regardless of the number of network changes. To speed up the calculation of a feasible condition set, it is possible to first solve individual wrong predictions against a “control” condition, thereby identifying conditions that cannot be reconciled with the network with the allowed modifications. We applied this strategy for the pre-processing of the *M*. *genitalium* data (see Results).

Furthermore, the number of binary variables can be reduced by a set of additional preprocessing steps. First, binary variables for changes to the network not allowed (such as reversibility changes to reactions strictly considered irreversible) should be constrained to zero. Second, we can consider a “supermodel” that encompasses the input model with all allowed reactions converted to reversible reactions and all reactions from the database of potential additional reactions. We can then reduce the number of binary variables further by (i) excluding all reactions that are blocked in this supermodel, (ii) constraining to zero the binary variables for the removal of reactions that are essential in this supermodel.

### Enumeration of alternative solutions

GlobalFit can optionally calculate a user-defined number *n* of alternative optimal or suboptimal solutions. The search for alternative solutions is executed using the integer cuts method. Thus, the complexity for each additional alternative solution is only increased through a single linear constraint. Consequently, the runtime for *n* alternative optimal or suboptimal solutions is approximately *n* times the runtime for a single optimum.

### Implementation and availability

We provide an implementation of GlobalFit, integrated with the *sybil* toolbox for constraint-based analyses [19], which runs in the R environment for statistical computing [36]. The source code and documentation is available free of charge from CRAN (http://cran.r-project.org/web/packages/GlobalFit/). The optimized models for *E*. *coli* and *M*. *genitalium* are provided as SBML files that can be read, e.g., by *sybil* [19] and the COBRA toolbox [37].

## Supporting Information

S1 Table Users of GlobalFit should choose appropriate penalties for proposed model changes based on the details of the network reconstruction and the proposed changes. As a starting point, this table list some suggested penalty values.
(PDF)

S1 Database To construct a database of potential additional reactions for the conservative application of GlobalFit to *M*. *genitalium*, we started from all reactions contained in metabolic networks provided by the BiGG database [24]. We then restricted this dataset to reactions that are catalyzed by enzymes with significant sequence similarity to the *M*. *genitalium* genome (BLAST e-value <10^−13^). We removed globally blocked reactions, *i*.*e*., those reactions of the database that were not able to carry any flux in a supernetwork containing all reactions. Reversible reactions were represented as two independent irreversible reactions, corresponding to forward and backward directions. The database is provided as a tab-delimited text file with three columns: reaction ID; stoichiometric equation; gene-protein-reaction association (GPR).
(TSV)

S2 Database To construct a database of potential additional reactions for the conservative application of GlobalFit to *E*. *coli*, we started from all reactions contained in metabolic networks provided by the BiGG database [24]. We then restricted this dataset to reactions that are catalyzed by enzymes with significant sequence similarity to the *E*. *coli* genome (BLAST e-value <10^−13^). We removed globally blocked reactions, *i*.*e*., those reactions of the database that were not able to carry any flux in a supernetwork containing all reactions. Reversible reactions were represented as two independent irreversible reactions, corresponding to forward and backward directions. The database is provided as a tab-delimited text file with three columns: reaction ID; stoichiometric equation; gene-protein-reaction association (GPR).
(TSV)

S1 Model The *M*. *genitalium* iPS189 models as modified by GlobalFit are supplied as an SMBL file, which can be read, e.g., by the *sybil* toolbox for *R* [19] or the COBRA toolbox for Matlab [37]. The two models differ only by their biomass reactions: “Biomass” for the non-conservative model; “Biomass_conservative” for the conservative model.
(XML)

S2 Model The *E*. *coli* iJO1366 model as modified by GlobalFit is supplied as an SMBL file, which can be read, e.g., by the *sybil* toolbox for *R* [19] or the COBRA toolbox for Matlab [37]. (XML)
