# Correction: Improved Metabolic Models for *E*. *coli* and *Mycoplasma genitalium* from GlobalFit, an Algorithm That Simultaneously Matches Growth and Non-Growth Data Sets

**DOI:** 10.1371/journal.pcbi.1005264

**Published:** 2016-12-09

**Authors:** The PLOS Computational Biology Staff

The funding statement for this article should read as follows:

We acknowledge financial support through the German Research Foundation DFG (GRK 1525, EXC 1028, and SFB 680). The funders had no role in study design, data collection and analysis, decision to publish, or preparation of the manuscript.

## References

[1] Hartleb D, Jarre F, Lercher MJ (2016) Improved Metabolic Models for *E*. *coli* and *Mycoplasma genitalium* from GlobalFit, an Algorithm That Simultaneously Matches Growth and Non-Growth Data Sets. PLoS Comput Biol 12(8): e1005036 doi:10.1371/journal.pcbi.1005036 27482704 10.1371/journal.pcbi.1005036 PMC4970803

