# Supplementary material for: Improved Metabolic Models for E. coli and Mycoplasma genitalium from GlobalFit, an Algorithm That Simultaneously Matches Growth and Non-Growth Data Sets
Source: PLoS Comput Biol. 2016 Aug 2;12(8):e1005036. doi: 10.1371/journal.pcbi.1005036 (PMC4970803; doi:10.1371/journal.pcbi.1005036)
Supplement: S1 Table — As a starting point, this table list some suggested penalty values. (PDF) [file pcbi.1005036.s001.pdf]

	Remove Reaction				Add Reaction	
	Non-Exchange reaction		Exchange reaction			
	With GPR	Without GPR	Defined media	Undefined media	With GPR	Without GPR
**Penalty**	3	1	10	0.1	1	5

	Reverse Reaction			Add Biomass metabolite		Remove Biomass metabolite
	Predicted reversibility			Included in other known biomass reactions		
	Without uncertainty	With uncertainty	None	Yes	No	
**Penalty**	1	5	10	1	20	1
